# The Cuisine of Hungary

**Published:** 1986-04

**Authors:** J. D. Davies

**Affiliations:** University Department of Pathology Bristol Royal Infirmary


**THE CUISINE OF HUNGARY**
**G. Lang**
**Penguin Books Ltd., Harmondsworth, Middlesex, 1985**
**pp. 495+xvi, ISBN 0 14 046681 9 Price £8.95**

Many cookery books are about the sort of food that you already know very well. Most of them are not in the least concerned with their origins, let alone with their cultural background. This book is not at all like that. It is a fascinating account of how 'Hungarian' food and dishes arose. It is about the amazing mixture of cultures, European history, and the everyday surprise that awaits anyone fortunate enough to stray through the Iron Curtain.

The book begins with a general account of goings-on since the 13th century in what is now Hungary. There are many delightful illustrations to help you on your way in this section. In any case the history is not too heavy. Just in case your culture appetite is too quickly sated, the culinary developments are discreetly hinted at, even at this stage. Mind you, satiation is unlikely with so many 18th century continental prints to ease your way.

A quick geographical whizz round Hungary, its vineyards, plants, people and places then sets you up for the serious stuff. To reassure you the set recipes are preceded by 'A Few Cautionary Notes'.

Any innocent who has gone to Hungary for a medical conference will know only too well why this section appears. Today it isn't mere 'Cautionary Notes' that you need. It is a cast-iron gastrointestinal tract, or the strong determination to escape yet another full 'touristic' menu. This book tells you what to think of when even more tempting cream, pieces of pork balls and steaming stews face you early at breakfast time. Forget the hotel; why not try the people's market place? Quieter things are on offer there. Even in Hungary – provided you can escape the official reception, breakfast or luncheon aimed to please the overweight Westerner – simple food really does exist. George Lang's book explains all this. No official host would dare, though!

Mind you Hungarian hospitality, even in this book, may put you off. There is a lot of cream and meat. Try the more simple dishes, i.e. anything with less than eight ingredients, first. Once in a while – if you fancy – go for something heavier; but not too often. That way you may possibly grow to like Hungarian dishes. How about this for an evening meal at home:

Caraway soup with garlic croutons
Cauliflower and goose liver
Sour potato
Stefania torte with Coffee

After that, I doubt if you would feel much like arguing as to whether it was good for you or not. In any case it would soon be bedtime for yourself, friends and relatives. Sleep well. But do try this book sometime before breakfast. You won't find too many recipes that you already know.

J. D. Davies
University Department of Pathology
Bristol Royal Infirmary

